# Association between Antihypertensive Drug Use and the Incidence of Cognitive Decline and Dementia: A Meta-Analysis of Prospective Cohort Studies

**DOI:** 10.1155/2017/4368474

**Published:** 2017-09-28

**Authors:** Guangli Xu, Feng Bai, Xin Lin, Qiongying Wang, Qiang Wu, Shougang Sun, Cheng Jiang, Qiang Liang, Bingren Gao

**Affiliations:** ^1^Department of Cardiology, Lanzhou University Second Hospital, Lanzhou 730030, China; ^2^Department of Urology, Lanzhou University Second Hospital, Lanzhou 730030, China; ^3^Department of Cardiac Surgery, Lanzhou University Second Hospital, Lanzhou 730030, China

## Abstract

**Background:**

Antihypertensive drug use is inconsistently associated with the risk of dementia, Alzheimer's disease, cognitive impairment, and cognitive decline. Therefore, we conducted a meta-analysis of available prospective cohort studies to summarize the evidence on the strength of these relationships.

**Methods:**

Three electronic databases including MedLine, Embase, and the Cochrane Library were searched to identify studies from inception to April 2017. Only prospective cohort studies that reported effect estimates with corresponding 95% confidence intervals (CIs) of dementia, Alzheimer's disease, cognitive impairment, and cognitive decline for antihypertensive drug use versus not using antihypertensive drugs were included.

**Results:**

We included 10 prospective cohort studies reporting data on 30,895 individuals. Overall, participants who received antihypertensive drugs had lower incidence of dementia (relative risk [RR]: 0.86; 95% CI: 0.75–0.99; *p* = 0.033), while there was no significant effect on the incidence of Alzheimer's disease (RR: 0.83; 95% CI: 0.64–1.09; *p* = 0.154), cognitive impairment (RR: 0.89; 95% CI: 0.57–1.38; *p* = 0.596), and cognitive decline (RR: 1.11; 95% CI: 0.86–1.43; *p* = 0.415). Further, the incidence of Alzheimer's disease might be affected by antihypertensive drug use in participants with specific characteristics.

**Conclusions:**

Antihypertensive drug use was associated with a significantly reduced risk of dementia, but not with the risk of Alzheimer's disease, cognitive impairment, and cognitive decline.

## 1. Introduction

Hypertension has been well documented as a modifiable risk factor for cardiovascular and cerebrovascular diseases [1], and its prevalence increases with advancing age [2]. In addition, changes in blood pressure are known to affect white matter hyperintensities, intima media thickness, and carotid artery atherosclerosis [3–5]. Recent evidence has shown that hypertension is the most important pathological factor for poor cognitive function [6–8]. The causes of dementia are complex including lifestyle, diet, age, brain injury, gender, diabetes mellitus (DM), coronary heart disease, hyperlipidemia during midlife, and tobacco use [9–11]. However, data on the effect of antihypertensive drug use on subsequent dementia, Alzheimer's disease, cognitive impairment, and cognitive decline are limited and inconclusive.

Several prospective studies have indicated that antihypertensive drug use may reduce the risk of dementia [12, 13], whereas other studies showed no association between the two [14–17]. Yasar et al. found that antihypertensive drug use may decrease the risk of Alzheimer's disease [18], while several other studies reported that antihypertensive drugs did not affect the incidence of Alzheimer's disease [12, 14, 15, 19, 20]. Given these conflicting findings, clarifying the treatment effects of antihypertensive drug use on dementia, Alzheimer's disease, cognitive impairment, and cognitive decline is particularly important in the hypertensive population. Herein, we attempted a large-scale meta-analysis of the available prospective cohort studies to determine the association between antihypertensive drug use and the risk of cognitive decline and dementia. Further, the treatment effects in specific subpopulations were also quantitatively elucidated in hypertensive patients.

## 2. Materials and Methods

### 2.1. Data Sources, Search Strategy, and Selection Criteria

This review was conducted and reported according to the Preferred Reporting Items for Systematic Reviews and Meta-Analysis Statement issued in 2009 (Checklist S1) [22].

Any prospective cohort study that examined the relationship between antihypertensive drug use and cognitive outcomes, including dementia, Alzheimer's disease, cognitive impairment, and cognitive decline, was eligible for inclusion. No restrictions were placed on language or publication status (published, in press, or in progress). We searched the MedLine, Embase, and Cochrane Library electronic databases for articles published through April 2017 and used “Alzheimer” or “dementia” or “cognition” or “executive functions” or “learning” or “visual perception” or “neuropsychology” or “psychomotor performance” and “antihypertensive” or “anti-hypertensive” and “cohort” or “prospective” or “longitudinal” as the search terms. We also conducted manual searches of the reference lists from all relevant original and review articles to identify additional eligible studies. The title, methods, population, design, exposure, and outcome variables of these articles were used to identify the relevant studies.

The literature search was independently conducted by two authors using a standardized approach. Any inconsistencies were settled by the primary author through consensus. The inclusion criteria were as follows: (1) the study had a prospective cohort design; (2) the study investigated the association between antihypertensive drug use and the incidence of dementia, Alzheimer's disease, cognitive impairment, and cognitive decline; and (3) the study reported effect estimates (risk ratio, hazard ratio [HR], or odds ratio [OR]) and 95% confidence intervals (CIs) for comparisons of hypertensive drug use and not using antihypertensive drugs. Studies were excluded if (1) the study had retrospective cohort or case-control design, (2) the comparison was conducted between hypertensive and normal participants, and (3) effect estimates could not be obtained or calculated.

### 2.2. Data Collection and Quality Assessment

The data collected included the first author's name, publication year, country, sample size, mean age, percentage of males, history of stroke, DM and cardiovascular disease (CVD), follow-up duration, effect estimate and its 95% CI, reported endpoints, and covariates in the fully adjusted model. For studies that reported several multivariable adjusted effect estimates, we selected the effect estimate that was maximally adjusted for potential confounders.

The Newcastle-Ottawa Scale (NOS), which is very comprehensive and has been partially validated for evaluating the quality of observational studies in meta-analysis, was used to evaluate the methodological quality [23]. The NOS is based on the following three subscales: selection (four items), comparability (one item), and outcome (three items). A “star system” (range: 0–9) was developed for assessment. The data extraction and quality assessment were independently conducted by two authors. Any discrepancy was independently examined and adjudicated by another author based on the original studies.

### 2.3. Statistical Analysis

We examined the relationship between antihypertensive drug use and the incidence of dementia, Alzheimer's disease, cognitive impairment, and cognitive decline based on the effect estimate (RR, HR, or OR) and its 95% CI published in each study. The RRs and 95% CIs for different categories of antihypertensive drugs were combined using the fixed-effects model [24]. Further, the random-effects model was employed to calculate summary RRs and 95% CIs for antihypertensive drug use versus not using hypertensive drugs [25]. Heterogeneity between studies was investigated using the *I*^2^ and *Q* statistics, and *p* < 0.10 was considered to indicate significant heterogeneity [26, 27]. Metaregression analyses were employed to explore any potential sources of heterogeneity for dementia and Alzheimer's disease based on the sample size, mean age, male percentage, history of stroke, DM, and CVD, and follow-up duration [28]. Subgroup analyses were conducted for dementia and Alzheimer's disease based on publication year, country, sample size, mean age, male percentage, history of stroke, DM, and CVD, follow-up duration, adjusted SBP and DBP or not, and adjusted BMI or not. We also performed a sensitivity analysis by removing each individual study from the meta-analysis [29]. Several methods were used to check for potential publication bias. Visual inspections of funnel plots for dementia and Alzheimer's disease were conducted. The Egger [30] and Begg [31] tests were also used to statistically assess publication bias for dementia and Alzheimer's disease. All reported *p* values are two-sided, and *p* < 0.05 was considered to be statistically significant. Statistical analyses were performed using STATA software (version 10.0, Stata Corporation, College Station, TX, USA).

## 3. Results

**Figure 1:**
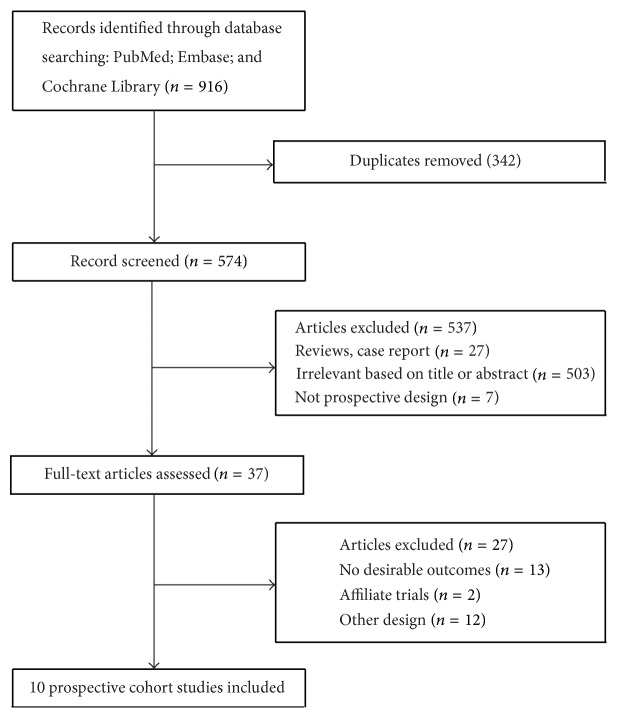
Flow diagram of the literature search and trial selection process.

The results of the study-selection process are shown in Figure 1. We identified 916 articles in our initial electronic search, of which 879 duplicates and irrelevant studies were excluded. A total of 37 potentially eligible studies were selected. After detailed evaluations, 10 prospective cohort studies were selected for the final meta-analysis [12–21]. A manual search of the reference lists of these studies did not yield any new eligible studies. The general characteristics of the included studies are presented in Table 1.

In the 10 included studies reporting data on 30,895 individuals, the follow-up duration was 2.2–32.0 years, while 302–6,537 individuals were included in each study. Five studies were conducted in the US, four were conducted in Europe, and one was conducted in Australia. The mean age ranged from 68.7 to 83.0 years, and the male percentage ranged from 0.0 to 100.0%. Further, the history of stroke ranged from 2.0 to 22.5%, the history of DM ranged from 6.6 to 100.0%, and the history of CVD ranged from 9.9 to 36.1%. The incidence of dementia was available in six studies, the incidence of Alzheimer's disease in six studies, the incidence of cognitive impairment in four studies, and the incidence of cognitive decline in two studies. Study quality was evaluated by the NOS scale, and a score of ≥7 was regarded as high quality. Overall, two studies had a score of 8, five studies had a score of 7, two studies had a score of 6, and one study had a score of 5.

**Figure 2:**
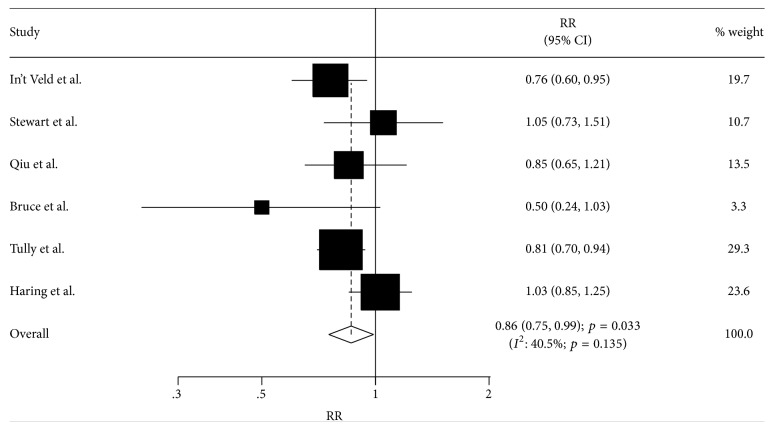
Association between antihypertensive drug use and the incidence of dementia.

A total of six studies reported an association between antihypertensive drug use and the incidence of dementia. The summary RR showed that antihypertensive drug use was associated with lower incidence of dementia (RR: 0.86; 95% CI: 0.75–0.99; *p* = 0.033, Figure 2), and moderate heterogeneity was detected (*I*^2^ = 40.5%; *p* = 0.135). Sensitivity analysis was conducted and the conclusions were affected by individually excluding the studies by In't Veld et al. (RR: 0.89; 95% CI: 0.76–1.04; *p* = 0.149), Qiu et al. (RR: 0.86; 95% CI: 0.73–1.02; *p* = 0.079), and Tully et al. (RR: 0.88; 95% CI: 0.73–1.06; *p* = 0.170) (Table 2).

**Figure 3:**
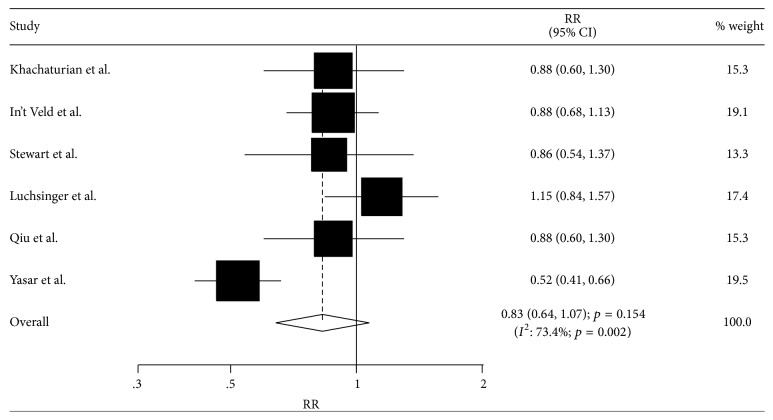
Association between antihypertensive drug use and the incidence of Alzheimer's disease.

A total of six studies reported an association between antihypertensive drug use and the incidence of Alzheimer's disease. There was no significant association between antihypertensive drug use and the incidence of Alzheimer's disease (RR: 0.83; 95% CI: 0.64–1.07; *p* = 0.154, Figure 3), and significant heterogeneity was observed (*I*^2^ = 73.4%; *p* = 0.002). In the sensitivity analysis, the study by Luchsinger et al., which did not specifically adjust for cardiovascular risk factors, was excluded. Subsequently, we noted that antihypertensive drug use significantly reduced the risk of Alzheimer's disease by 23% as compared to participants without antihypertensive drug use (RR: 0.77; 95% CI: 0.60–1.00; *p* = 0.047, Table 3).

**Figure 4:**
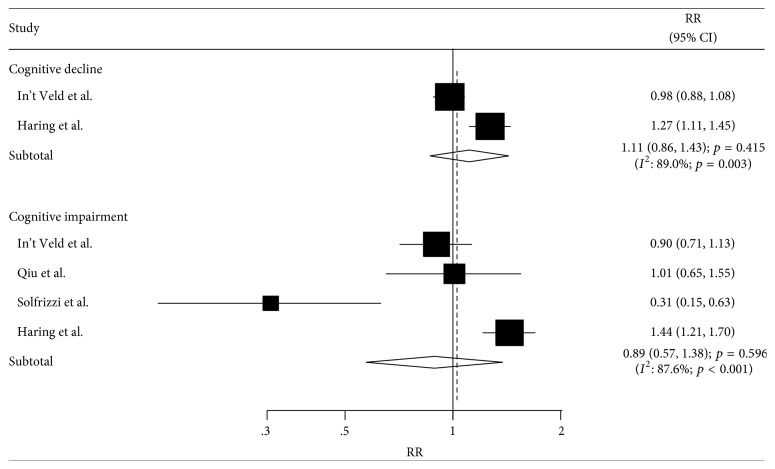
Association between antihypertensive drug use and the incidence of cognitive impairment and cognitive decline.

The number of studies available for each outcome was four studies and two studies for cognitive impairment and cognitive decline, respectively. The summary results for cognitive impairment and cognitive decline indicated that the comparison of antihypertensive drug use with participants without antihypertensive use showed no significant difference in the incidence of cognitive impairment (RR: 0.89; 95% CI: 0.57–1.38; *p* = 0.596, Figure 4) and cognitive decline (RR: 1.11; 95% CI: 0.86–1.43; *p* = 0.415).

Heterogeneity testing showed potential heterogeneity for dementia and Alzheimer's disease. Therefore, metaregression analyses were conducted based on sample size, mean age, male percentage, history of stroke, DM, and CVD, and follow-up duration. These factors did not significantly contribute to the incidence of dementia and Alzheimer's disease.

Subgroup analyses suggested that antihypertensive drug use was associated with lower incidence of dementia if the study was published before 2010 (RR: 0.82; 95% CI: 0.67–1.00; *p* = 0.050), the study was conducted in other countries (RR: 0.79; 95% CI: 0.71–0.89; *p* < 0.001), mean age was < 80 years (RR: 0.78; 95% CI: 0.69–0.89; *p* < 0.001), male percentage was < 50% (RR: 0.84; 95% CI: 0.73–0.97; *p* = 0.021), history of stroke was < 10% (RR: 0.82; 95% CI: 0.72–0.93; *p* = 0.003), history of CVD was < 20% (RR: 0.88; 95% CI: 0.77–1.00; *p* = 0.042), follow-up duration was < 5 years (RR: 0.70; 95% CI: 0.60–0.96; *p* = 0.019), and the study was adjusted for SBP and DBP (RR: 0.80; 95% CI: 0.70–0.90; *p* < 0.001). Further, participants who received antihypertensive drugs were associated with reduced risk of Alzheimer's disease if the study was published in 2010 or later (RR: 0.52; 95% CI: 0.41–0.66). No other significant differences for Alzheimer's disease were detected between antihypertensive drug use and participants without antihypertensive drug use (Table 4).

**Figure 5:**
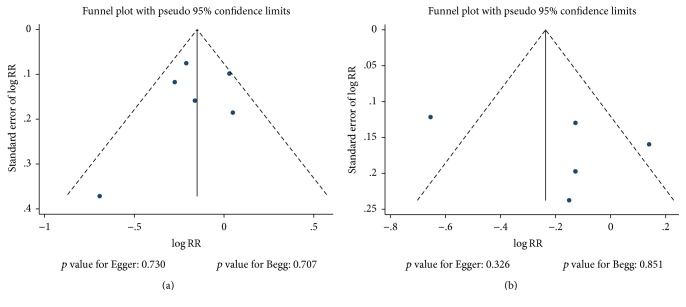
Funnel plots for dementia (a) and Alzheimer's disease (b).

Review of the funnel plots could not rule out the potential for publication bias for dementia and Alzheimer's disease (Figure 5). The Egger and Begg test results showed no evidence of publication bias for dementia (*p* value for Egger: 0.730; *p* value for Begg: 0.707) and Alzheimer's disease (*p* value for Egger: 0.326; *p* value for Begg: 0.851).

## 4. Discussion

Our meta-analysis was based on prospective cohort studies and explored all possible correlations between antihypertensive drug use and the outcomes of dementia, Alzheimer's disease, cognitive impairment, and cognitive decline. This comprehensive quantitative study included 30,895 individuals from 10 prospective cohort studies with a broad range of populations. The findings suggested that antihypertensive drug use was associated with reduced incidence of dementia, with no significant effect on Alzheimer's disease, cognitive impairment, and cognitive decline. Further, the findings of stratified analyses might differ based on different characteristics.

A previous meta-analysis based on eight randomized controlled trials and six cohort studies found that antihypertensive drug use was associated with a significantly reduced risk of vascular dementia and other dementia, but not with the incidence of Alzheimer's disease, cognitive impairment, and cognitive decline [32]. The findings of this study are in contrast with the findings of subgroup analysis in the previous meta-analysis and also indicate that antihypertensive drug use has a beneficial effect on dementia, but not on Alzheimer's disease, cognitive impairment, and cognitive decline. The reason for this could be that several studies with similar results on cognitive decline and dementia [13, 17, 18, 21] were published after the previous meta-analysis. The limitation of the previous meta-analysis was that it combined randomized controlled trials and prospective cohort studies, while the treatment effects of antihypertensive drugs in participants with specific characteristics were not examined. Further, several data abstracted were inconsistent with the original articles. Therefore, we conducted this quantitative meta-analysis to evaluate any potential relationships between antihypertensive drug use and the outcomes of dementia, Alzheimer's disease, cognitive impairment, and cognitive decline.

Most of our findings were in agreement with a large cohort study conducted in Netherlands [12]. This prospective study included 6,416 individuals and found that participants who received antihypertensive drugs at baseline had a lower incidence of dementia, and this reduction was introduced by vascular dementia (RR: 0.30; 95% CI: 0.11–0.99). Although the incidence of Alzheimer's disease reduced by 13%, it was not statistically significant. Tully et al. suggested that non-dihydropyridine calcium channel blockers and loop diuretics were strongly and independently associated with a reduced risk of dementia. Further, the changes in SBP were not the primary mechanism for the incidence of dementia when patients received antihypertensive drugs [13]. The findings of this study also indicated that antihypertensive drug use significantly reduced the risk of dementia. The possible reason could be that antihypertensive drugs might have a neuroprotective action in addition to directly lowering the blood pressure. Further, the imbalance of intracellular calcium might play an important role in neurodegeneration and cause cell pathology and apoptosis [33, 34]. Finally, perturbed calcium homeostasis might play an important role in mitochondrial dysfunction and oxidative stress in dementia patients, especially for patients correlated with presenilin-1 mutations [35]. Calcium influx leads to cytoskeleton alterations similar to neurofibrillar tangles in patients with dementia [36].

There was no significant difference between antihypertensive drug use and participants without antihypertensive drugs and the risk of Alzheimer's disease, cognitive impairment, and cognitive decline. However, several studies included in our analysis reported inconsistent results. Yasar et al. indicated that use of diuretics, angiotensin-1 receptor blockers, and angiotensin-converting enzyme inhibitors was independently associated with lower incidence of Alzheimer's disease in participants with normal cognition, while, in mild cognitive impairment patients, diuretic use significantly reduced the risk of Alzheimer's disease [18]. Further, Haring et al. indicated that antihypertensive drug use in patients with uncontrolled blood pressure showed higher incidence of cognitive decline and cognitive impairment [17]. However, Solfrizzi et al. found that angiotensin-converting enzyme inhibitors might reduce the risk of mild cognitive impairment [21]. The possible reasons could be that the levels of sodium intake might be associated with the degree of blood pressure, independent of blood pressure changes, and the level of sodium loading increases oxidative stress and endothelial dysfunction, which could promote vascular aging [37, 38].

Subgroup analysis suggested that antihypertensive drug use was associated with a reduction in dementia if the study was published before 2010 or conducted in other countries, mean age was <80 years, male percentage was <50%, history of stroke was <10%, history of CVD was <20%, follow-up duration was <5 years, and the study was adjusted for SBP and DBP. Antihypertensive drug use was associated with a reduced risk of Alzheimer's disease if the study was published in 2010 or later. These conclusions indicated that antihypertensive drugs might play an important role in primary prevention of cognition impairment. Further, these relationships in several subsets might vary due to sample size. Therefore, we showed a relative result and provided a synthetic and comprehensive review.

Three strengths of this study should be highlighted. First, only prospective cohort studies were included, which would eliminate selection and recall bias that could be detrimental in retrospective observational studies. Second, the large sample size allowed us to quantitatively assess the association of antihypertensive drug use with the risk of cognition, and thus our findings are potentially more robust than those of any individual study. Third, the relationships between antihypertensive drug use and the risk of cognition in specific subsets and patients with different characteristics were examined.

The limitations of this study are as follows: (1) the adjusted models differed across the included studies, and these factors might play an important role in the development of dementia, Alzheimer's disease, cognitive impairment, and cognitive decline; (2) in a meta-analysis of published studies, publication bias is an inevitable problem; and (3) the analysis used pooled data (individual data were not available), which restricted us from performing a more detailed relevant analysis and obtaining more comprehensive results.

## 5. Conclusions

The findings of this study suggested that antihypertensive drug use might play an important role in the incidence of dementia, but not in the incidence of Alzheimer's disease, cognitive impairment, and cognitive decline. Future large-scale prospective studies should focus on specific populations to analyze the primary or secondary prevention of dementia, Alzheimer's disease, cognitive impairment, and cognitive decline.

## Figures and Tables

**Table 1:** Baseline characteristics of the studies included in the systematic review and meta-analysis.

Study	Publication year	Country	Sample size	Mean age (yrs)	Percentage male (%)	History of stroke (%)	History of DM (%)	History of CVD (%)	Reported outcomes	Follow-up periods (yrs)	Adjusted factors	NOS score
Khachaturian et al. [19]	2006	USA	3297	74.1	41.8	4.2	11.2	11.1	AD	3.0	Age, sex, education, APOE *ε*4 alleles, DM, cholesterol, MI, and stroke	8
In't Veld et al. [12]	2001	Netherlands	6416	68.7	41.3	2.3	10.0	NA	Any dementia, AD, VD, CI, and CD	2.2	Age, gender, DBP and SBP, DM, stroke, BMI, baseline MMSE, smoking, education, living situation, and PAD	7
Stewart et al. [14]	2009	USA	1890	83.0	100.0	2.0	32.0	NA	Any dementia, AD	32.0	Age	7
Luchsinger et al. [20]	2005	USA	1138	76.2	30.2	NA	20.3	28.9	AD	5.5	Age, gender, education, APOE-ɛ4 allele, and ethnicity	6
Qiu et al. [15]	2004	Sweden	947	80.9	22.9	NA	NA	NA	Any dementia, AD, and CI	6.0	Age, sex, education, MMSE score, vascular morbidity, functional dependence, and APOE *ε*4 alleles	7
Bruce et al. [16]	2008	Australia	302	75.7	48.3	22.5	100.0	36.1	Any dementia	7.6	Crude	5
Tully et al. [13]	2016	France	6537	77.2	38.0	4.9	14.2	10.6	Any dementia	8.4	Sex, center, education, BMI, stroke, hypercholesterolemia, DM, center, CKD, APOE-ɛ4, SBP, and DBP	7
Solfrizzi et al. [21]	2013	Italy	873	72.0	53.0	6.6	11.6	17.0	CI	3.5	Age, gender, education, smoking status, type 2 DM, CAD, serum creatinine level, apolipoprotein B to apolipoprotein A1 ratio, history of stroke, and hypertension	6
Haring et al. [17]	2016	USA	6426	65–79	0.0	*NA*	6.6	16.5	Any dementia, CI, and CD	9.1	Age, race, education, WHI menopausal hormone therapy arm, baseline 3MSE, alcohol, smoking status, physical activity, DM, BMI, depression, total energy intake, and history of CVD	8
Yasar et al. [18]	2013	USA	3069	78.6	53.8	2.9	9.0	9.9	AD	6.1	Age, sex, education, income, CVD, BMI, SBP, DBP, and MCI	7

*Note.* MMSE: mini-mental state examination; PAD: peripheral atherosclerotic disease; CKD: chronic kidney disease; CAD: coronary artery disease; CI: cognitive impairment; CD: cognitive decline; DM: diabetes mellitus; MI: myocardial infarction; DBP: diastolic blood pressure; SBP: systolic blood pressure; BMI: body mass index; PAD: peripheral arterial disease; CVD: cardiovascular disease.

**Table 2:** Sensitivity analysis for dementia.

Excluding study	RR and 95% CI	*p* value	Heterogeneity (%)	*p* value for heterogeneity
In't Veld et al.	0.89 (0.76–1.04)	0.149	43.2	0.134
Stewart et al.	0.84 (0.73–0.97)	0.021	44.2	0.127
Qiu et al.	0.86 (0.73–1.02)	0.079	52.3	0.078
Bruce et al.	0.88 (0.77–1.00)	0.042	35.8	0.183
Tully et al.	0.88 (0.73–1.06)	0.170	45.1	0.122
Haring et al.	0.81 (0.73–0.91)	<0.001	0.7	0.402

**Table 3:** Sensitivity analysis for Alzheimer's disease.

Excluded study	RR and 95% CI	*p* value	Heterogeneity (%)	*p* value for heterogeneity
Khachaturian et al.	0.82 (0.61–1.11)	0.206	78.3	0.001
In't Veld et al.	0.82 (0.59–1.14)	0.237	77.6	0.001
Stewart et al.	0.83 (0.61–1.11)	0.205	78.6	0.001
Luchsinger et al.	0.77 (0.60–1.00)	0.047	67.1	0.016
Qiu et al.	0.82 (0.61–1.11)	0.206	78.3	0.001
Yasar et al.	0.93 (0.80–1.09)	0.380	0.0	0.696

**Table 4:** Subgroup analyses for dementia and Alzheimer's disease.

Outcomes	Group	RR and 95% CI	*p* value	Heterogeneity (%)	*p* value for heterogeneity	Intersubgroup heterogeneity
Dementia	*Publication year*					
	2010 or after	0.91 (0.72–1.15)	0.409	73.4	0.052	0.432
	Before 2010	0.82 (0.67–1.00)	0.050	25.4	0.259	
	*Country*					
	USA	1.03 (0.87–1.23)	0.697	0.0	0.927	0.011
	Other	0.79 (0.71–0.89)	<0.001	0.0	0.584	
	*Sample size*					
	≥1000	0.88 (0.76–1.03)	0.119	51.7	0.102	0.492
	<1000	0.73 (0.45–1.17)	0.186	42.0	0.189	
	*Mean age (years)*					
	≥80.0	0.93 (0.73–1.18)	0.543	0.0	0.386	0.052
	<80.0	0.78 (0.69–0.89)	<0.001	0.0	0.422	
	*Male percentage (%)*					
	≥50.0	1.05 (0.73–1.51)	0.792	—	—	0.267
	<50.0	0.84 (0.73–0.97)	0.021	44.2	0.127	
	*History of stroke (%)*					
	≥10.0	0.50 (0.24–1.04)	0.062	—	—	0.077
	<10.0	0.82 (0.72–0.93)	0.003	10.1	0.329	
	*History of DM (%)*					
	≥20.0	0.78 (0.38–1.59)	0.489	68.7	0.074	0.949
	<20.0	0.86 (0.72–1.02)	0.090	60.8	0.078	
	*History of CVD (%)*					
	≥20.0	0.50 (0.24–1.04)	0.062	—	—	0.140
	<20.0	0.88 (0.77–1.00)	0.042	35.8	0.183	
	*Follow-up duration periods (years)*					
	≥5.0	0.89 (0.76–1.04)	0.149	43.2	0.134	0.243
	<5.0	0.70 (0.60–0.96)	0.019	—	—	
	*Adjusted SBP and DBP*					
	Yes	0.80 (0.70–0.90)	<0.001	0.0	0.647	0.052
	No	0.94 (0.77–1.14)	0.510	32.1	0.220	
	*Adjusted BMI*					
	Yes	0.86 (0.72–1.02)	0.090	60.8	0.078	0.866
	No	0.85 (0.63–1.16)	0.315	38.8	0.195	

Alzheimer's disease	*Publication year*					
	2010 or after	0.52 (0.41–0.66)	<0.001	—	—	<0.001
	Before 2010	0.93 (0.80–1.09)	0.380	0.0	0.696	
	*Country*					
	USA	0.81 (0.54–1.21)	0.309	82.6	0.001	0.210
	Other	0.88 (0.71–1.09)	0.238	0.0	1.000	
	*Sample size*					
	≥1000	0.82 (0.61–1.11)	0.206	78.3	0.001	0.560
	<1000	0.88 (0.60–1.30)	0.517	—	—	
	*Mean age (years)*					
	≥80.0	0.87 (0.65–1.17)	0.366	0.0	0.941	0.470
	<80.0	0.82 (0.57–1.17)	0.268	83.6	<0.001	
	*Male percentage (%)*					
	≥50.0	0.64 (0.39–1.04)	0.074	71.9	0.059	<0.001
	<50.0	0.94 (0.80–1.11)	0.478	0.0	0.556	
	*History of stroke (%)*					
	≥10.0	—	—	—	—	—
	<10.0	0.75 (0.55–1.02)	0.068	73.1	0.011	
	*History of DM (%)*					
	≥20.0	1.05 (0.80–1.37)	0.724	3.0	0.310	0.026
	<20.0	0.73 (0.50–1.06)	0.100	80.9	0.005	
	*History of CVD (%)*					
	≥20.0	1.15 (0.84–1.57)	0.381	—	—	0.001
	<20.0	0.66 (0.40–1.10)	0.114	80.6	0.023	
	*Follow-up duration periods (years)*					
	≥5.0	0.81 (0.54–1.21)	0.309	82.6	0.001	0.210
	<5.0	0.88 (0.71–1.09)	0.238	0.0	1.000	
	*Adjusted SBP and DBP*					
	Yes	0.68 (0.40–1.13)	0.135	88.6	0.003	0.004
	No	0.97 (0.80–1.17)	0.718	0.0	0.598	
	*Adjusted BMI*					
	Yes	0.68 (0.40–1.13)	0.135	88.6	0.003	0.004
	No	0.97 (0.80–1.17)	0.718	0.0	0.598	
